# Satisfaction and Quality of Life of Families Participating in Two Different Early Intervention Models in the Same Context: A Mixed Methods Study

**DOI:** 10.3389/fpsyg.2021.650736

**Published:** 2021-04-27

**Authors:** Sebastià Verger, Inmaculada Riquelme, Sara Bagur, Berta Paz-Lourido

**Affiliations:** ^1^Childhood, Technology, Education and Diversity Research Group, Institute of Research and Innovation in Education (IRIE), Palma, Spain; ^2^Department of Applied Pedagogy and Psychology of Education, University of the Balearic Islands, Palma, Spain; ^3^Institute of Health Sciences Research (IUNICS-IdISBa), University of the Balearic Islands, Palma, Spain; ^4^Department of Nursing and Physiotherapy, University of the Balearic Islands, Palma, Spain

**Keywords:** early intervention, empowerment, patient satisfaction, family, child development disorders

## Abstract

Early intervention is developed following different types of service organization, which in turn require different professional and family roles. The aim of this study was to compare the perceived satisfaction and family quality of life amongst families receiving early intervention developed at centers in comparison to those receiving the routines-based early intervention in families’ homes, that is a family centered intervention in ecological environments. Under a transformative paradigm, a mixed methods design was used, using the Consumer Report Effectiveness Scale (CRES-4) and the Beach Center Family Quality of Life Scale (FQOLS) and two focus groups as data collection instruments. The sample comprised 166 parents in the quantitative phase and 16 parents in the qualitative phase. Results showed that families receiving routines-based early intervention had greater satisfaction with the service although both groups showed similar scores for family quality of life in most of the analyzed domains. Three dimensions were identified throughout the qualitative phase: problem solving, professional team and service organization. Both the models analyzed have an impact on family quality of life and parents are in general satisfied. Strengths and weaknesses were found related to the problem-solving process, the role of the professional team, family empowerment and the service’s organization. The areas requiring further development are the effective training of professionals focused on family practices, the exchange of information with the family and a more participatory organization that takes parent’s perspectives into consideration. The value given to a combined model is another aspect highlighted in this study, as well as the need for a more agile assessment period to avoid unnecessary delays.

## Introduction

Early intervention is the set of interventions for children and their families to provide support, improve personal development, strengthen family skills, and promote the inclusion of the family and the child, through experiences and learning opportunities plus the support and resources provided by caregivers (GAT, 2005; Dunst et al., 2019). The conceptualization of early intervention as well as the organization of services oriented toward children and families have gone through different stages during the last decade (Guralnick, 2001). In the sixties, early childhood intervention was mainly influenced by the biomedical paradigm, focused on the mental, physical, or sensory deficiencies and therefore, services where addressed to rehabilitating the children’s different affected areas, with little room for family involvement in the process (Dunst, 1998; Rouse, 2012; Romero et al., 2015; Belda, 2016; Díaz, 2019).

The seventies decade was significant for the current early intervention paradigm, influenced by theoretical perspectives such as the transactional model of Sameroff and Chandler (1975), the ecology of human development model of Bronfenbrenner (1979), the theory of structural cognitive modifiability of Feuerstein (1980) or Leal’s (1999) theory of family systems where family is understood as a complex system with its own unique characteristics that evolve depending on their members’ needs (Leal, 2008; Minuchin, 2012). The approval of the Convention on the Rights of the Child was another milestone for the development of early childhood services because the full development of children was seen as a collective responsibility of all citizens (UN, 1989; Veerman, 1992). The importance and potential value of a worldwide commitment to provide comprehensive early intervention services for children at risk of or with identified disabilities is relevant but differs amongst countries and regions worldwide (Guralnick and Albertini, 2006).

Early intervention requires formal and informal support networks, considering the family system as a whole (Dunst, 2000; Davis and Gavidia-Payne, 2009), because one of the main objectives of early childhood intervention is to offer professional support addressed to a better family wellbeing, as well as responding to the temporary or permanent needs of children with developmental disorders or at risk of developing them (Dunst, 2000; GAT, 2005; Burger, 2010; UNESCO, 2016). Family quality of life has historically received limited attention despite its crucial role in shaping early childhood development (Summers et al., 2005), but this construct is important for assessing the possible impact of services and support for families. Family quality of life is understood as the subjective perception of family well-being. It refers to the family’s dynamic sense of well-being, collectively and subjectively defined and informed by its members, in which individual and family-level needs interact (Smith-Bird and Turnbull, 2005; Giné et al., 2008; Zuna et al., 2010).

Early intervention services may be developed in early intervention centers, but also in natural environments such as homes, schools, or leisure spaces, now seen as contexts to promote development. In Spain, early childhood intervention is generally carried out in early intervention centers and, to a lesser extent, at home or in other settings (García-Sánchez et al., 2018). Early intervention centers are defined as specialized centers with suitable infrastructure and a multi-disciplinary professional team, responsible for providing integral attention to the underage and to their families (GAT, 2018). These professionals, belonging to disciplines such as physiotherapy, psychology, social work, or speech therapy, collaborate with each other from an interdisciplinary approach (Evans, 2017). In this model of early intervention children have access to individualized attention in accordance with the child’s particular needs including families’ perspectives and collaboration when needed (Millaì, 2005; Belda, 2016).

The routines-based early intervention is a relatively recent approach (McWilliam, 2010). It focuses on achieving functional outcomes, namely a child’s independence, social relationships with others, and parents’ satisfaction with daily routines, by providing the children with learning opportunities in naturally occurring contexts and systematically using collaboration and coaching to set functional goals and implement service plans with the family. This model goes beyond other strategies of family-centered intervention (Rosenbaum et al., 1998). Professionals work in a transdisciplinary way (Russell et al., 2008) so that each family is in contact with one professional who is the one who makes the regular home visits and follows the intervention progress. This means that the professional is the one who visits the child and family in their own environment interacting with them and with the resources available in those particular spaces (Rouse, 2012; Martínez and Calet, 2015). Recent studies highlight the advantages of early interventions developed in natural environments, such as increased communication with families (Fingerhut et al., 2013), or a better understanding regarding the children’s disability or needs in comparison with those developed in specific early childhood intervention centers (Pighini et al., 2014).

Therefore, as in other Spanish regions, the early services in Mallorca are experiencing a transition process between both intervention models. Considering the significant public investment in terms of financial and personal resources, it is essential to evaluate the quality of early intervention services, particularly because research on this area is still scarce (Torres-Samuel and Vaìsquez-Stanescu, 2015; López et al., 2018; Romero-Galisteo et al., 2019). The variables determining the quality of early intervention services have been identified in previous studies (Jemes-Campaña et al., 2019), and family satisfaction has been found as a predictor variable that should be considered to evaluate the services (GAT, 2005; Millaì, 2005; Bruder and Dunst, 2015; Romero et al., 2015; Belda, 2016).

This study aims to assess the families’ satisfaction and quality of life regarding the early intervention services. The identification of families’ perceptions about the received services is essential to determine those aspects that are best valued, providing the feedback needed to ensure quality services (Davis and Gavidia-Payne, 2009; Pighini et al., 2014; Dias and Cadime, 2019; Hughes-Scholes and Gavidia-Payne, 2019). Previous studies show that an adequate assessment of early services should consider the parents’ opinions about the professional team, their professional training, coordination, or development of the intervention plan (Romero et al., 2015). Family satisfaction is governed by the family’s perception of the professional, personalized attention, effective communication, knowledge of family rights, team coordination or the intervention plan (Martínez and Martínez, 2013; Romero et al., 2015; Hughes-Scholes and Gavidia-Payne, 2019). Family satisfaction is directly related to family empowerment (Botana and Peralbo, 2014; Pighini et al., 2014; Mas et al., 2019).

## Materials and Methods

### Context of the Study and Design

The study was developed in Mallorca (Balearic Islands, Spain) as a part of a broader research project developed in different stages. In Spain, early intervention is addressed to children from 0 to 6 years old and their families. It is a decentralized public/subsided service organized at regional level. Children are usually referred from pediatric health services but also from early childhood education centers when a disability, developmental or language delay is identified or at risk of developing. All families entering the early childhood intervention program have to pass through diverse protocolized evaluations prior to being redirected to any of the providers selected based on the family’s residence. As mentioned previously, although for decade providers only offered early intervention in multidisciplinary centers, in recent times some providers are also offering routine-based early intervention in natural environments. As developed in the specific geographic context of this study, in addition to the differences related to the location where the intervention takes place, there is also a change in the way of working of the professionals involved. While in the centers an intervention is developed from an interdisciplinary approach, in natural or ecological environments a single professional intervenes with a transdisciplinary approach. All the professionals that participate in this model have received specific training to work in a transdisciplinary way and with a routine-based approach.

The transformative paradigm (Mertens, 2007; Shannon-Baker, 2016) was considered the theoretical framework of this study. Using this perspective means paying particular attention to issues of power, privilege, and voice. All these aspects take place in the field of early childhood intervention, where in addition to the limitation of public resources, the diverse organizations developed imply different roles for professionals and families, which requires the voices of families to be heard.

Taking into consideration the complexity of early intervention, an explanatory sequential mixed-methods design was chosen in this study (Teddlie and Tashakkori, 2009; Uprichard and Dawney, 2016; Plano-Clark, 2019). The mixed methodology has great potential to study the same phenomenon from different perspectives (Schoonenboom and Johnson, 2017) and this complementarity seeks clarification of the results from one method with the results from the other one, but also data triangulation (Greene et al., 1989; Chaves, 2018). The integration of both perspectives covers the entire research and requires flexibility and dynamic strategies to complete the set of results (Fielding, 2012; Akerblad et al., 2020). In this case, an exploratory quantitative phase was followed by a qualitative phase.

### Participants

A total number of 166 families participated in this study, of which 77 were receiving early intervention at centers (EIC model) and 89 were receiving family centered early intervention in natural or ecological environments (EINE model).

### Instruments

Consumer Report Effectiveness Scale (CRES-4). This questionnaire, initially used in the Consumer Reports (1995) assesses parents’ satisfaction with the therapeutic intervention, with four items evaluating parents’ satisfaction with the intervention and their perception of effectiveness in three domains: Satisfaction, Problem solving, and Emotional change perception. A global score intends to disclose the perceived intervention effectiveness. Higher scores indicate higher perception of effectiveness. This questionnaire is considered to be useful in combination with other validated instruments (Seligman, 1995). The validated Spanish version of the questionnaire was used (Feixas et al., 2012).

Beach Center Family Quality of Life Scale (FQOLS). This questionnaire contains 25 items assessing family importance and satisfaction ratings within five domains: Family interaction, Parenting, Emotional well-being, Physical/material well-being, and Disability-related supports (Summers et al., 2005; Hoffman et al., 2006; Hu et al., 2011). For the purpose of this study, only the Satisfaction dimension was used. Parents had to score each item on a Likert scale from 1 (very unsatisfied) to 5 (very satisfied). A total score was also computed, with higher scores indicating higher satisfaction with family quality of life. Reliability was assessed and reported (Summers et al., 2005; Hoffman et al., 2006). Cronbach’s alpha for the FQOL subscales on Satisfaction ratings was 0.88, and test-retest reliability was examined in satisfaction responses for each of the FQOL subscales. All correlations were significant at the 0.01 level or beyond (df from 59 to 63). For the satisfaction dimension, the correlations between time points were 0.75 for Family Interaction, 0.71 for Parenting, 0.76 for Emotional Well-being, 0.77 for Physical / Material Well-being, and 0.60 for Disability-Related Support. The validated Spanish version of the scale was used (Verdugo et al., 2009). The psychometric properties of this instrument have been demonstrated in previous studies (Balcells-Balcells et al., 2016).

Regarding qualitative data collection procedures, two focus groups were developed. Focus groups allow the exploration of specific issues, provide an environment for participants to share their thoughts and feelings and can assist with the validation of experiences by other group members or research strategies (Kitzinger, 1996). Group size is usually from six to nine people and have been widely used in early childhood intervention (Brotherson and Goldstein, 1992; McLachlan, 2005). The development of the focus groups in this study followed the recommendations related to the environment, the interview process, and the role of the researchers in order to obtain valuable information from the participants and create productive participation dynamics (Krueger and Casey, 2014).

### Recruitment, Eligibility, and Procedure

The recruitment of families for this study was carried out amongst users of early childhood intervention providers in Mallorca, Balearic Islands, Spain. Families were contacted through email or phone and were invited to participate in this study. Those who agreed to participate were asked to fill out a short questionnaire to verify the following inclusion criteria regarding their children (a) from 0 to 6 years old, and (b) receiving early childhood intervention for at least 6 months. Once this was verified, participants were invited to fill out a sociodemographic questionnaire (see Table 1) and the scales.

**TABLE 1:** Sociodemographic characteristics of families participating in EINE and EIC models.

	*n*_EINE_ = 89	*n*_EIC_ = 77
Mother’s age	36.50 (5.98)	37.37 (4.99)
Father’s age	39.46 (7.61)	39.36 (4.76)
Child’s age	3.85 (1.27)	3.75 (1.29)
Number of siblings	1.86 (0.78)	1.95 (0.78)
**Children attending early intervention services (*n*)**		
One	80	71
Two	7	4
**Parents work situation (*n*)**		
Both employed	18	26
One employed	64	46
Both unemployed	7	5

Parents were also informed about the qualitative phase. Those interested in participating were contacted to gather further information about their family characteristics. Through convenience sampling (Koerber and McMichael, 2008), parents with different profiles were selected (employment status, place of residence, gender, income level, number of children, number of children with disabilities, child development problem and level of support). Two focus groups were developed, one with parents attending early intervention centers and one with parents receiving routines-based early intervention in their homes. Each focus group included eight parents (seven mothers and one father) with different socio-demographic conditions, but similar between both groups. Regarding the participants’ employment situation, both groups were made up of families where only one of the parents was employed (7) or both employed (1). Regarding their children’s limitations, both groups included parents of children with severe and mild psychomotor retardation (3), language delay (3), and intellectual disability (2).

Regarding the focus group of parents attending early intervention centers, three of them attended physiotherapy services with their children, three to psychology services and two to speech therapy. Regarding the routines-based early intervention focus group, four participants had the psychologist as the reference professional, while two had the physiotherapist and another two had the speech therapist.

The interview script included questions aimed at obtaining the families’ perspectives on the early intervention service in a comprehensive way, trying to obtain details about the aspects that underlie their satisfaction, the perceived impact on children and families, their opinion on the role of professionals, their empowerment level, and quality of family life. Strengths and weaknesses of the service and the room for improvement were also asked. Each meeting lasted around 90 min and was moderated by one researcher whilst another served as an observer, taking notes on the development of the sessions and non-verbal communication. Both researchers were familiar with focus group interviewing procedure. Data collection ceased when saturation on major themes was reached and no new information emerged (Patton, 2002). To ensure rigor (Mays and Pope, 1995), besides the different strategies described above, both researchers present during the interview met shortly after leaving the interview room to document impressions and reflections so as to improve the accuracy and thoroughness of the descriptions. The sessions took place in a separated area of the early intervention center, to ensure the confidentiality and anonymity of the participants. The interviews were digitally recorded and transcribed.

### Analysis

To analyze the quantitative data, the statistical analysis was carried out, comparing the sociodemographic and clinical characteristics with the *t* test for independent measures and with the chi-square test for categorical variables. The means and standard deviations of the variables were calculated. A normal distribution of quantitative variables (*p* > 0.05) was found with the Kolmogorov–Smirnov test and group comparison tests (ECCN and ECCC) were performed. The IBM SPSS Statistics 19.0 package was used, with an importance level set at *p* < 0.05.

Data from the quantitative phase was helpful to configure the participants’ selection, interview script elaboration and data interpretation in the qualitative phase. For the analysis of the qualitative data, once the verbatim was obtained, a content analysis was performed using the NUDIST software. Throughout the qualitative data analysis process, the researchers met in person to discuss the emerging codes and categories, and to resolve interpretation discrepancies.

### Ethical Issues

The study was carried out under the ethical and legal considerations required in research. Regarding the quantitative phase, the questionnaires were anonymous and participation in the study was completely voluntary. Regarding the qualitative phase, all participants signed the informed consent and data protection was ensured. Confidentiality was guaranteed, so the qualitative findings are reported with the pseudonym Parent, accompanied by EINE_n, if it is the group that receives early intervention in natural environments, or by EIC_n, if it is the group that attends early intervention centers, where *n* = 1,…,8, refers to each particular participant. Ethical considerations have also been taken into account during the transcription phase removing names or sensitive data that could allow the participants, children, or professionals’ identification. The approval of the Research Ethics Committee of the Balearic Islands (CEIB / IB) was obtained, assigning the file code 3182/16 PI.

## Results

The two family groups revealed similar results for the sociodemographic variables (both *t* < 0.92, both Chi-square < 6.62, both *p* > 0.09), as well as the clinical ones (*t* = 0.35, both Chi-square < 7.49, both *p* > 0.06). Both the diagnosis (Chi-square = 12.99, *p* = 0.043) and the professionals who provided the practice (Chi-square = 28.06, *p* < 0.001) varied between one group and the other.

The reason for getting early intervention support or diagnosis were language delay (EINE = 36%, EIC = 27.3%), maturational delay (EINE = 14.6%, EIC = 14.2%), prematurity (EINE = 7.9%, EIC = 0%), genetic syndrome (EINE = 6.7%, EIC = 10.38%), mixed disorder (EINE = 5.6%, EIC = 10.38%), motor delay (EINE = 3.4%, EIC = 7.79) and others. Regarding the type of intervention received, there are differences, since 48% of those involved in the EIC model required the services of more than one professional, which is not the case in the transdisciplinary EINE model where this only happens in 12.4% of cases, as consistent with the characteristics of each model.

The results derived from the Consumers Reporting Efficacy Scale (Table 2) indicated that parents who received EINE had greater scores in satisfaction [*t*(1,104) = 2.38, *p* = 0.02], problem solving [*t*(1,97) = 2.54, *p* = 0.018] and intervention efficacy [*t*(1,97) = 2.50, *p* = 0.019] than parents who received EIC intervention. However, no significant differences were found in the perception of emotional change [*t*(1,140) = −0.06, *p* = 0.95].

**TABLE 2:** Consumer Report Effectiveness Scale (CRES-4).

	EIC model					EINE model				
	*N*	Minimum	Maximum	Mean	Std. Deviation	*N*	Minimum	Maximum	Mean	Std. Deviation
Satisfaction	76	,00	100,00	81,052	25,901	30	,00	100,00	64,0000	35,77709
Problem solving	75	60,00	100,00	93,8667	9,84932	24	,00	100,00	75,0000	35,99517
Emotional change	84	38,00	100,00	62,1548	12,10545	58	37,50	100,00	62,2845	12,27682
Total	75	162,50	287,50	237,8333	33,39296	25	62,50	287,50	201,5000	70,01488

The Beach Center Family Quality of Life Scale (Table 3) offered high total scores (*X* > 97.5 ± 17.18/13.47) and did not differ between the two groups [*t*(1,154) = 0.02, *p* = 0.98]. Statistically significant differences were found in two domains: disability-related supports [*t*(1,158) = −3.97, *p* < 0.001] and physical well-being [*t*(1,158) = 3.23, *p* = 0.002]. These results show greater satisfaction with supports for EIC participants and with physical well-being in the EINE group. In the domains of family interaction, parenting, and emotional well-being, no statistically significant differences were found (*t* < 1.21, *p* > 0.23).

**TABLE 3:** Beach Center Family Quality of Life Scale (FQOLS).

	EIC model					EINE model				
	*N*	Minimum	Maximum	Mean	Std. Deviation	*N*	Minimum	Maximum	Mean	Std. Deviation
Family interaction	85	12,00	30,00	24,1059	5,05452	76	8,00	30,00	24,4211	4,97799
Parenting	88	7,00	30,00	22,5682	5,30191	76	5,00	30,00	22,8947	5,24488
Emotional well-being	88	7,00	20,00	13,9886	3,54120	76	6,00	20,00	13,3289	3,40740
Physical well-being	84	10,00	25,00	18,9762	3,99993	76	11,00	20,00	17,3158	2,37324
Disability supports	84	11,00	20,00	17,5476	2,58077	76	11,00	25,00	19,5132	3,55713
Total	80	57,00	125,00	97,5375	17,17515	76	63,00	125,00	97,4737	13,46450

The analysis of the questionnaires revealed the need to delve into the questions of satisfaction and family quality of life in the qualitative phase. Parent’s perspectives obtained through the focus groups interviews were classified in three dimensions and eight categories (see Table 4).

**TABLE 4:** Qualitative dimensions and categories.

Dimension	Category	Information
1. Problem solving	1.1 Professional support	Professional treatment, case monitoring, interpersonal skills, relationship, and family availability
	1.2 Child’s needs	Attention, personalization, adaptation, and interaction
	1.3 Empowerment	Participation, orientation, information, autonomy, and involvement
2. Professional Team	2.1 Training	Intervention, specialization, and professionals
	2.2 Goals	Guidelines and routines
	2.3 Teamwork	Coordination, team, and transdisciplinar interprofessional
3. Organization of the service	3.1 Case evaluation	Diagnosis, delays, duration, and process
	3.2 Sessions	Duration, schedule, and frequency

In general, families from both groups reported high satisfaction with regards to the services received, although this satisfaction was low regarding the assessment period prior to enter the early childhood intervention program. This period can last several weeks or even months. They described this time as distressing, tiring and bureaucratic, considering this as a delay to early intervention: “*You start with the social worker, then with the psychologist or speech therapist and, in our case, we got very disoriented. It is very slow”* (Parent EINE_4). In addition, another complaint was: “*The amount of tests that we have been subject to and that take such a long time”* (Parent EIC_1), which shows another aspect of low satisfaction amongst families. Both aspects, the large number of tests and the various professional visits required, were not always seen as a process aimed at providing a better service, but very often as time that is taken away from early intervention activities that would otherwise benefit their children.

But regarding their experience in early childhood intervention services, as said before, most of the comments referred to positive impacts in the children and family, illustrating their satisfaction with being finally enrolled in the program *“we are lucky, we are finally getting early intervention to our child”* (Parent EINE_3). Problem solving was particularly valued in EINE interventions. This model promotes the professional adaptation to the family’s dynamics and also the identification of the inconveniences that exist at home. Participants highlight professional support as a crucial aspect for the appropriate development of EINE. Family needs are overcome as the sessions progress because the professional in this model observes the difficulties close by as they follow-up on the case. This has an impact on their emotional well-being, as described by the Parent EINE_1: “*The professional’s talks and advice, the guidelines to follow with our child and the tasks to do at home give us peace of mind and resolve doubts*.” In addition to the professional intervention and individualized case monitoring, parents valued their ability to adapt to the home context, their interpersonal skills and attitude and the close relationship with the family: *“We value the personal approach toward us, it offers us a sense of being widely supported”* (Parent EINE_3).

For parents attending the EIC model, going to the early intervention center also has positive effects on their quality of life, since this allows them to talk to other parents, which is seen as useful for problem solving and getting emotional support from peers. As an example of this perception Parent EIC_7 stated: *“It is also good for us to come to the center, where we can speak to others. When my child was a baby it would have been nice for him to have had early intervention at home, and also would have been more comfortable for us. But now I prefer to come here. It’s good for us to come here, to go out for a while, talk, disconnect.”* In the EIC focus group, direct intervention with the child is particularly valued whereas interventions with parents, as receiving emotional support, are not always seen as needed: *“We come here for the children, we are fine. I like this professional because he/she works a lot with the child, we come here for the child, not for ourselves”* (Parent EIC_2).

Both groups of parents positively valued the early intervention provided, although they refer to differences in the practices developed. The EINE practices are described as mutual listening and guidance based on specific family needs. Different parents’ discourses detail the importance of carrying out the intervention at home because the professional can really observe the difficulties in context: *“They see every little thing that occurs at home and how problems emerge. If the professional had not come home, my child would not put into practice all that he/she has learnt”* (Parent EINE_8). Despite this, the general consideration was that intervention should have an emphasis on the child. In cases in which the professional does home visits without the child being present, for example, to give strategies to the parents, this situation was experienced negatively, because they feel they are being evaluated, instead of the child: *“The professional teaches us the tasks to develop with our child, but it should be the child who is assessed, her/his stage should be checked before continuing with new exercises. But in my case, my child is at school on many of the days the professional comes to our home”* (Parent EINE_2).

In general, the perceptions of EIC participants regarding the developmental improvement of their children is positive. In the early intervention center, professionals can see how far the child is progressing and therefore adapt strategies and resources to each case. In addition, the child is more task focused, since “*At home the child might be more confused because it is his/her own environment”* (Parent EIC_4). Reducing the distractions and focusing on the treatment is seen as beneficial for all parts involved in therapy. Parent EIC_3 stated the following: *“It is necessary that the professional bond with the child in their treatment spaces. If they come home, we are all there, and I feel that it is necessary for the child to recognize a space to be alone with the professional.”*

Regarding empowerment, parents receiving EIC do not see capacity-building to be so relevant to their parental role. Being trained in simple tasks to allow continuity of care with their children was in general seen as necessary but having a more active role in early intervention strategies with the child was in some cases experienced with anguish. This was verbalized during the group interview: “*It seemed that I was the patient (…) As a parent, I see that it is me who is getting the intervention and it should be my child. From an ethical point of view, this shouldn’t be done”* (Parent EIC_5). In contrast, families receiving EINE described positive impacts on what they considered to be empowered. They referred to the abilities, creative strategies, or resources they learn from professionals, which can later be developed autonomously: *“They have taught me how to lead, manage and better understand my child’s situation, I participate more, we do the tasks using games with my child”* (Parent EINE_5). The satisfaction of learning how to intervene with their children encourages parents to put solutions to work on their children’s benefit.

But family empowerment was also described in less satisfactory manner. For some, their parental role goes too far, forcing them to make what they consider to be professional decisions: *“Imagine that you have a headache and the doctor, instead of giving you the appropriate medication, asks you: what do you want to take to get rid of your headache, or what you are going to do for your headache? You are the doctor, I don’t know”* (Parent EINE_7).

Obtaining accurate information from professionals was seen as a crucial aspect influencing parent satisfaction in both groups. But EINE participants pointed out that sometimes they are being offered too much information, too many options and therefore, the freedom to decide becomes the stress to choose the correct option: *“Professionals sometimes give us too much free rein, it should be more agile: Tell me clearly what is best for me, even if you make a mistake”* (Parent EINE_4).

Satisfaction with the professional team was also very good in both groups, but particular issues emerged for each group. Parents who receive EINE perceived the need for further professional training, but this was not identified in the case of EIC parents. In their own words, *“I have noticed that sometimes, they ask you many questions, as if they had doubts. Maybe this is due to the fact that this model is more innovative”* (Parent EINE_7). In addition, this group considered that since they only have one professional during the home visits, sometimes very specific doubts arise that must be consulted with another professional, which causes delays to an agile resolution of the problem.

The goal setting processes was described differently. Parents attending EIC explained that the professional proposed goals for the child, and they follow the same goals, ensuring continuity of care. This is remarked as positive in the EIC group: *“The professional sets the goals, what we have to do at home,… I am happy,”* Parent EIC_8. In contrast, in the routine-based early intervention model, goals are established on parents’ concerns basis. Together with professional guidance, parents participate in the establishment of goals regarding their child, but also for the whole family, and this is also seen as positive: “*The professional also establishes goals for me. Works with the child and with me. For example, giving me ideas of what I should work on by myself”* (Parent EINE_1).

Regarding teamwork, parents attending the EIC services are very satisfied with the coordination among professionals: “*If they consider that another professional should see the child or talk to us, we are referred to”* (Parent EIC_3). In the EINE model, considering that the intervention is carried out by just one professional, interviewed parents valued the transdisciplinary knowledge of other disciplines. The Parent EINE_6 states: “*I was very surprised that I could not only ask questions about speech therapy, but that I could raise questions about issues such as diet or behavior.”* In any case, they were satisfied with the good communication amongst professionals, necessary in order to respond to specific queries. Parent EINE_3 summarizes that “*Whenever you have a problem, they give you guidelines to start working with. In my case, my professional of reference is a psychologist, and is the one who comes home, but he/she brings me materials that the speech therapist recommends to us, so that my child can work at home.”*

The service’s organization is the aspect where parents see room for improvement, for EINE parents, in particular. The duration of the session is stipulated to be 1 h, regardless of the case. This is seen as negative, asking for flexibility on this issue and also regarding the frequency of sessions. For example, Parent EINE_6 indicates that “*If the session were longer, he would have time for improvement in the different states my child goes through. More sessions per week are needed. I think one session is not enough.”* On the other hand, for another participant a session every 2 weeks was seen as more than enough. Therefore, the general demand is an adaptation of the duration and frequency of the service taking into account parent’s opinions.

The EIC group was in general satisfied with the fixed schedule and timing to visit the early intervention center, but they would prefer to be able to choose a combined model. They define this model as *“Going to the center and having them (professionals) come home too”* (Parent EIC_2). The early intervention service that families received (EINE or EIC) seemed to depend on an organizational decision based on professional evaluations and geographical aspects, but the parents’ speeches suggested that their opinions were not sufficiently considered. Having the possibility of choosing the model, or having a combined model, is another aspect where groups converged, and it was seen as an improvement to ensure that the service fits the particular needs of each family. However, that possibility was also seen as a utopia, because in his own words: *“I don’t think it is possible to organize it in this way, it will require too many professionals*” (Parent EIC_6).

## Discussion

This study shows that families are in general satisfied with the early intervention received, both those who attend EIC services and those who receive EINE services. Both groups reported several positive aspects with regards to the services received in the qualitative phase, which were consistent with the exploratory findings in the quantitative phase. But another issue of coincidence was the low satisfaction regarding the assessment period prior to entering the early childhood intervention program. Taking into consideration findings in the qualitative phase, the fact of finally being enrolled in a public/subsided service despite the waiting list and delays entering the early intervention program, could be one of the reasons influencing such satisfaction.

Findings in the quantitative phase, reported similar quality of life scores in the areas of family interaction, parenting style, or emotional well-being, but they differ in the sphere of physical well-being (higher for EINE parents) and in the support received (better valued in EIC families). The qualitative results show a variety of issues influencing this perceived satisfaction and family quality of life, classified in three categories: problem-solving, professional team and service organization.

In order to carry out EINE intervention professionals have to develop their professional activity from a transdisciplinary and family-centered approach and this requires professional adaptation to the family’s upbringing style. Families interviewed were generally satisfied with this process, describing the importance of relational and participatory practices. It is known that direct observation of routines as well as family dynamics favor the analysis of the deficits and the family’s potential (Mas et al., 2019), which was much appreciated by participants in this study. This requires generic professional competencies and interpersonal skills in order to allow the development of effective interventions, providing support in a systemic perspective and providing responses according to the family’s emotional needs. Professional beliefs about family strengths, their decision-making or the active participation of family members are crucial in the development of goals aimed at increasing capacities and adopting new skills (Espe-Sherwindt, 2008; Mas et al., 2019). In this study, the increased family participation in the EINE group compared to those receiving EIC services was seen as positive, although this should not diminish the importance of the child’s progress assessment or getting expert professional opinion when needed. On the contrary, EIC parents valued the specialized professional’s perspective, and therefore the parent’s role is described as a resource for continuity of care following the professional’s advice. The early childhood intervention developed in centers relates to some of the characteristics of the biomedical model based on the child’s impairment or delay, although involving families in basic and operational issues that have an impact on the children’s development. This way of working seems to be known to these parents and they are satisfied with it, especially with the agile interdisciplinary work carried out in the centers, which already means an important progress compared to the evaluation period comprising multiple visits before entering the early intervention program. In fact, EIC families reported better scores for the perceived support, and this was one of the elements described during the interviews.

In the case of EINE participants, these particularly pointed out that sufficient time is not always dedicated, and visits should be more frequent, a result similar to the one found by Gavidia-Payne et al. (2015). This creates controversy with some of the fundamentals of the EINE model that gives priority to guidance regarding how the intervention should be carried out by families, establishing goals together and follow-up on phases of intervention. Therefore, EINE families should be well informed about this particular early intervention model, since without such information they will expect the intervention to be developed only based on their children’s needs and not on those of the family system (Epley et al., 2010; Fox et al., 2015).

Findings show that the support the professional provides to the parents by way of resources, information or specialized therapy is particularly important in the case of EIC, which coincides with previous works by Martínez and Martínez (2013) where the information received was one of the aspects best valued by the parents. The exchange of information is one of the basic pillars of all kinds of interventions (Balcells-Balcells et al., 2016; Marco et al., 2018). However, oral transmission does not imply that family members understand and have sufficient strategies to integrate the process and incorporate new guidelines into their daily routines (Escorcia-Mora et al., 2018). This study shows different parents’ perspectives on learning new skills to support their children. The relational and participatory practices of EINE are directly identified with the development of new coping strategies and the learning of new family capacities (Mas et al., 2019). Training parents means incorporating more components of personal control, knowing the resources and alternatives, and learning to attribute potentialities to personal circumstances (Dempsey and Dunst, 2004). The professional-family relationship must be strongly linked to parental self-efficacy beliefs (Rouse, 2012).

Learning new strategies and skills is seen as positive in order to address the child’s condition as well as to be empowered to develop creative solutions to problems. But EINE parents’ discourses also show the consideration of empowerment as a free decision-making process for the family, missing a professional counseling role. This different perception of families regarding their empowerment and preparedness to assume an active role in early intervention was also found in previous studies (Rodger et al., 2012; Mayorga et al., 2015). In fact, the required level of family empowerment is considered to be one of the challenges for the development of EINE services (Espe-Sherwindt, 2008; Cañadas, 2012; Rodger et al., 2012; Dias and Cadime, 2019) because it is dependent on family variables for its correct development, such as the characteristics of each family or the place of residence (Fingerhut et al., 2013; Pighini et al., 2014). Empowerment means offering opportunities and experiences for family members to be more competent, self-sufficient, and independent (Leal, 2008), based on the capacities that the family possesses or must acquire in order to control problems and improve their interpersonal and social life (Dunst et al., 1988; Dempsey and Dunst, 2004). Although some families were very satisfied with their perceived empowerment, other families have not experienced it in the same way, which shows the need of further research in this particular area.

The importance of a close relationship between professionals and families was seen as positive and is consistent with other findings in the literature regarding the EINE model. It is not enough to be affectionate or pleasant, but the professional must establish a relationship of trust with a balance of power, so that families understand what the process is and what power they have based on their capacities (Dunst, 2000; Rouse, 2012; Escorcia-Mora et al., 2018; Dunst et al., 2019). Participatory practices in early childhood intervention are in fact considered added value for everybody involved in the process, families and professionals (Espe-Sherwindt, 2008; Escorcia-Mora et al., 2016).

EINE services include interventions at home or in other natural settings provided by one reference professional working from a transdisciplinary perspective. It is whom coordinates with the rest of the team in order to establish the appropriate intervention plan for each case, as described for the EINE model (Minard, 2018). Therefore, authors describe that there is a kind of liberation from the disciplining role, where professional experience and knowledge come together to be applied by all team members (Cumming and Wong, 2012). The results of this study indicate that there is a need in the EINE services to train professionals to carry out this practice safely. The exchange of experiences or skills between professionals is currently a feared task (Evans, 2017) due to the scarce transdisciplinary training and education.

The establishment of intervention goals was different in both groups, since they follow different models, for the EINE model the development of common goals together with the families, is crucial (Cañadas, 2012; Pighini et al., 2014). The results detail that despite interacting with the family within an ecological framework, EIC professionals do not build goals jointly with the parents to the extent that they are being developed in the EINE services. Although it is considered that the professional goal setting process as developed in EIC may drive to a lack of understanding of the families as a whole (Dempsey and Dunst, 2004), this was nevertheless seen as positive, and families felt comfortable letting the professionals develop the intervention without their participation since they are experts in their disciplines.

The organization of the service was valued differently in both groups, and while EIC parents were in general very satisfied, there is room for improvement, in words of EINE participants. Session duration, schedule flexibility or timing are factors that tend to hinder positive satisfaction for EINE participants, which differs from Martínez and Martínez’s (2013) findings. For Cañadas (2012), policies and service management should prioritize the quality of the sessions rather than the quantity. Therefore, there is a demand from parents to be heard regarding the organization and service planning in order to, for example, make interventions coincide with the whole family instead of developing diverse sessions with different family members due to an insufficient coordination.

As mentioned previously, both groups were satisfied with the intervention’s effectiveness regarding the children’s needs but differ regarding the location where it should be developed. EINE parents place importance on listening, observing during daily routines and identifying real deficits that occur when the intervention is developed in natural settings. These aspects are, in fact, the areas of greatest satisfaction in relation to the service received. This is consistent with previous studies, that conclude that intervening in the child’s natural context has greater benefits for their development than practices in the center, where communication is less regular (Fingerhut et al., 2013; Pereira and Serrano, 2014) and the parents’ understanding of the disorder is not as thorough (Pighini et al., 2014). However, the EIC group highlighted the importance of having a space isolated from interruptions and addressed specifically to intervention, where the professional is able to observe the child in his/her own environment. Nevertheless, participants from both groups refer to the benefits of having a combined model including the assessment period and the intervention program. A combined intervention would require different roles for parents and professionals, and its appropriateness should be identified at the beginning of the intervention (Gavidia-Payne et al., 2015), as well as implemented with the adequate professional resources. However, participants consider that this option is far from being developed, precisely due to the increase in necessary resources.

In summary, the perception of family members regarding early intervention services is a fundamental component to adapt and implement quality practices. Research can assess the necessary methodological changes, as well as the impacts in children and families and the overall efficacy of the service (Davis and Gavidia-Payne, 2009; Pighini et al., 2014; Dias and Cadime, 2019; Hughes-Scholes and Gavidia-Payne, 2019). This research shows that families from both models find aspects of satisfaction and wellbeing in early childhood intervention. As described before, there are different elements that explain their satisfaction, but one aspect of coincidence is the value given to a combined model, the possibility of participating in the service organization and a more agile intervention during the assessment period to avoid unnecessary delays. Findings in this study highlight the need for further research including policy makers, professional perspectives, as well as children’s voices (Mas et al., 2018).

## Limitations of the Study

This study has some limitations that should be considered. One of them is related to the fact that presents information and perceptions collected from families involved in early intervention services but does not evaluate the impact of those interventions on children’s health and development. Regarding the used methodology, data obtained in the exploratory quantitative phase was limited but was relevant to the qualitative phase, focused on detecting the reasons for such satisfaction. The number of focus groups developed was less than initially expected. A greater number of groups could have provided more information and favored triangulation of the data. Even so, the selection of participants, the script preparation with data obtained in other stages of the study and the level of participation during the sessions still allowed for a great variety of contributions which tended to coincide. Regarding the sample in the qualitative phase, despite the fact different families’ profiles were considered in the recruitment process, the involvement of fathers was low. Another limitation that must be considered and that can interfere in the opinions of the families is the child’s diagnosis or level of health impairment. Findings in this study provide knowledge about families’ satisfaction regarding two different early intervention services, but transferability of this findings to other contexts should consider the diversity of administrative organizations, structures and services involved in early intervention.

## Conclusion

Both the models analyzed have an impact on family quality of life and parents are in general satisfied. Strengths and weakness were found related to the problem-solving process, the role of the professional team, family empowerment and the service’s organization. Areas for development are the effective training of professionals focused on family practices, the exchange of information with the family and a more participatory organization taking into consideration parent’s perspectives. The value given to a combined model is another aspect highlighted in this study, as well as the need for a more agile assessment period to avoid unnecessary delays.

However, it is necessary to continue with the study to give other voices a chance to be heard and give a more complete picture considering not only families, but also policy makers, professionals, and children. Further refinement of policies and advances in effective early intervention practices will require research but also the corresponding efforts to bring findings up to scale.

## Data Availability Statement

The datasets presented in this article are not readily available because anonymous quantitative data can be requested. Verbatim from qualitative research includes participant’s confidential data, so it will not be shared. Requests to access the datasets should be directed to BP-L, bpaz@uib.es.

## Ethics Statement

The studies involving human participants were reviewed and approved by Research Ethics Committee of the Balearic Islands. The patients/participants provided their written informed consent to participate in this study.

## Author Contributions

SV and BP-L have contributed equally and share senior authorship, what refers to the project design and development. IR and SB have contributed to the quantitative phase of the study. All authors have participated in the manuscript preparation.

## Conflict of Interest

The authors declare that the research was conducted in the absence of any commercial or financial relationships that could be construed as a potential conflict of interest.
